# Unraveling personality in mood disorders: the role of big five personality traits in Han Chinese women with bipolar and unipolar depression

**DOI:** 10.3389/fpsyt.2025.1596956

**Published:** 2025-06-13

**Authors:** Yuchen Lin, Jieying Gan, Zonglin He, Ting Yang, Xiuhua Wu, Qi Zhu, Zhaoyu Gan

**Affiliations:** ^1^ Department of Psychiatry, The Third Affiliated Hospital of Sun Yat-sen University, Guangzhou, China; ^2^ The First Clinical School of Guangzhou Medical University, Guangzhou, China; ^3^ Department of Orthopedics & Traumatology, Li Ka Shing Faculty of Medicine, The University of Hong Kong, Pokfulam, Hong Kong SAR, China

**Keywords:** big five personality, mood disorder, depression, bipolar disorder, personality traits

## Abstract

**Background:**

Personality traits are closely related to psychiatric disorders, but their role in distinguishing major depressive disorder (DD) from bipolar disorder (BD) remains unclear. The Big Five Inventory (BFI) is widely used to assess personality, yet findings on trait differences between BD and MDD and their relationship with clinical features are inconsistent. This study examines how personality traits vary across mood disorders and their associations with clinical features.

**Objective:**

This study aims to investigate how Big Five personality traits differ between major depressive disorder (MDD) and bipolar disorder (BD) and to explore their associations with clinical characteristics, including illness severity, hospitalization history, and comorbidities, in a sample of Han Chinese women.

**Methods:**

A cross-sectional analysis was conducted on BD, MDD, and healthy participants. Personality traits were assessed using the Chinese version of BFI-44, and clinical data including depression severity (HAMD scores), hospitalization history, and comorbidities were collected. Multiple regression, multivariate analysis of variance (MANOVA) and structural equational model (SEM) were used to examine group differences and associations between personality and clinical variables.

**Results:**

BD and MDD patients exhibited higher neuroticism than healthy controls (p<0.001). BD patients had lower agreeableness than MDD (β=-0.14, p=0.04), while extraversion did not significantly differentiate the disorders. BD-I patients had higher openness than BD-II (M=2.78, p=0.02) and higher conscientiousness than BD-NOS (M=3.51, p=0.03). Higher openness correlated with physical comorbidities (β = 0.19, p = 0.01), and neuroticism was strongly linked to depression severity (β = 0.26, p < 0.001).

**Conclusions:**

Neuroticism is a key marker of psychiatric illness, while agreeableness distinguishes BD from MDD. The associations between personality, illness severity, and treatment engagement highlight their potential clinical relevance and need for further study.

## Introduction

Mood disorders are characterized by prolonged disturbances in emotional states. The most common mood disorders are Bipolar Disorder (BD) and Major Depressive Disorder (MDD) (1). These disorders are chronic and widespread psychiatric conditions, associated with high risks of suicide (2) and relapse (3), making them significant contributors to global disease burden (4). Their etiology is complex and multifactorial (5), such as genetic predisposition, inflammation, and personality traits (1). However, the relationship between personality traits and mood disorders remain unclear (6).

According to Santesteban-Echarri et al. (7), personality interacts with mood disorders in a variety of ways. Their research outlines six models that describe how personality can be linked to the onset, development, and prognosis of mood disorders. An important perspective suggests that premorbid personality traits may serve as vulnerability markers for mental illness and could potentially predict different treatment outcomes. Studies have shown that certain personality traits are associated with vulnerability to mood disorders, such as high neuroticism, low extraversion, and conscientiousness with depression and anxiety (8). In addition, a meta-analysis provided strong evidence that extraversion is negatively correlated with MDD (9). A longitudinal study also found that neuroticism predicts anxiety and depression, confirmed by other cross-sectional studies (10, 11). This suggests that personality traits are a “default setting” that a person notices and reacts to life events, leading to negative affect and potentially mood disorders.

Personality also influences the clinical characteristics of mood disorders (12) and predicts its course (6, 10). In a therapy study (13), MDD patients were divided into two classes: one is the vulnerable class, which had high neuroticism and low extraversion and conscientiousness, and the other the resilient class, which had moderate neuroticism, extraversion, and high agreeableness and conscientiousness. Being in the resilient class was an indicator of faster and more successful treatment. A similar approach was used in patients with bipolar disorder, for whom higher vulnerability predicted a higher long-term morbidity index (6). Additionally, it is found that low conscientiousness and high neuroticism is positively correlated with suicidality (14).

Mood disorders can change personality both temporarily and permanently. Studies have shown that psychiatric symptoms, particularly in patients with bipolar disorder (15), influence response quality in self-rated instruments (16) and that simultaneously evaluating the severity of symptoms could reduce these effects. A similar study (17) from almost three decades ago was conducted in patients with euthymic bipolar disorder and in recovered patients with unipolar depression. Hamilton Depression Rating Scale (HAMD) scores were assessed to ensure that results were not affected by the severity of depression. Neurosis, which is thought to be most strongly associated with the severity of depression, did not correlate with HAMD scores, suggesting that studying euthymic and recovered patients minimizes potential confounding effects of the condition. Although “temporary” is a key-word here, as the core personality remains stable over time, persistent mood disorders can lead to changes in behavior and self-perception, ultimately leading to a reinforcement cycle (18). For example, having high neuroticism can increase vulnerability to stress and aggravate mood disorders (14), while mood disorders reinforce maladaptive personality patterns, leading to further worsening.

Previous studies that investigated the relationship between personality and mood disorders have identified various problems that may affect their conclusions that have not been systematically addressed. First of all, mood disorders are not homogenous. Mood disorders include MDD and BD, while BD can be further classified into Type 1 (BD-I) and Type 2 (BD-II). Studies have found that extraversion and agreeableness differ significantly between BD and MDD patients (10, 11). Extraversion was found to persist in BD throughout the illness course, but not in MDD (10). Our previous study (19) showed patients with recurrent MDD have lower extraversion, conscientiousness, openness to experience, and higher neuroticism. BD patients also exhibit elevated neuroticism, which is even more extreme in patients with borderline personality disorder (BPD) (20). BD patients who have been previously misdiagnosed as MDD have higher extraversion (21), which is similar to the case in BD subtypes, where BD-II patients had higher neuroticism and lower extraversion (22). These findings suggest that there is indeed personality diversity in mood disorder. In addition, clinical characteristics such as disease onset, physical or mental comorbidities, course of the disease, symptomatic characteristics and familial genetic burden all increase heterogenicity and complicate the interaction with personality traits. However, the above-mentioned clinical characteristics have rarely been systematically investigated in studies. Secondly, demographic features such as age, gender, and ethnicity were found to significantly contribute to personality. Previous research has shown that between early adulthood and middle age, agreeableness and conscientiousness tend to increase, whereas neuroticism and openness tend to decrease, while extraversion remains relatively stable (23–25). In terms of gender differences, the most consistently reported findings indicate that women tend to score higher in traits such as agreeableness, neuroticism, and openness to experience, while men tend to score higher in extraversion and openness to experience (26). Ethnicity has been found to moderate gender differences in certain personality traits (26, 27). Finally, variations in personality scales could contribute to differing conclusions across studies, as various models have been developed to simplify the complexity of personality traits. Among these, the Big Five Personality traits are the most widely applied (28). The majority of the variance in human behavior can be explained by the five broad domains: extraversion, openness to experience, neuroticism, agreeableness, and conscientiousness (29, 30).There are several measures to assess the Big Five Personality traits, including the 240-item NEO Personality Inventory (NEO-PI-R) (31), the shortened NEO Five Factor Inventory (NEO-FFI) (32), the International Personality Item Pool (IPIP) (33), and the 44-item Big Five Inventory (BFI-44) (34). However, the first two are proprietary instruments, which limits their availability for research (33, 35). In addition, some personality measures are quite extensive, such as the NEO-PI-R, which consists of 240 items. Empirical evidence suggests that lengthy scales can lead to participant discouragement, fatigue, and inattention, increasing the risk of bias and missing data due to non-completion (36). Therefore, a brief and freely available instrument, the BFI-44, was developed by John et al. in 1991 (37) and has demonstrated reliability, convergent validity and strong self-peer agreement (38, 39). Although shorter scales such as the Ten-Item Personality Inventory (TIPI) (40) and BFI-10 (41) have been introduced more recently, they are limited by weaker psychometric properties (42).

A recent study (12) examined personality in MDD and BD using the Eysenck Personality Questionnaire (EPQ), but its narrow scope and binary format limit sensitivity. The BFI offers a broader, more granular assessment, including conscientiousness and openness. Additionally, Li et al. focused on state-based comparisons but did not explore personality’s predictive value for mood fluctuations or long-term outcomes.

Building on this, we hypothesize that personality differences exist not only across mood disorder categories (BD vs. MDD) but also in relation to specific clinical characteristics. To test this, we will assess and compare the personality traits of BD and MDD patients using the Chinese version of the BFI, focusing on Han Chinese women. By controlling for potential confounders, this study aims to provide a more precise understanding of the relationship between personality and mood disorders and identify clinical characteristics associated with specific personality traits.

## Method

The sample consisted of 252 BD patients, 185 MDD patients and 103 healthy controls. Patients were recruited from the inpatient and outpatient psychiatry ward of the Third Affiliated Hospital of Sun Yat-sen University between October 2018 and October 2022. A total of 64 healthy controls (HCs) were recruited from the local community during the study period through voluntary participation. Additionally, 39 age- and sex-matched HCs were recruited from the community in December 2024. All participants were Han Chinese women. The Han ethnicity is the predominant ethnic group in China, accounting for approximately 91% of the national population (43). This selection aimed to minimize ethnic variability in personality expression. They were aged 16 to 65, with at least a junior high school education to ensure their ability to comprehend and complete all psychological assessments. Each participant provided written informed consent. Participants aged under 18 were required to provide written informed consent from their guardian. The patients had to meet the following criteria: (a) fulfill the diagnostic criteria for bipolar disorder of any type or major depressive disorder (MDD); and (b) have no comorbid organic mental disorder. Patients who were too ill to cooperate with the required assessments were also excluded. HCs were screened for mental disorders using the Chinese version of the Structured Clinical Interview for DSM-IV-TR Axis I Disorders (SCID-I), non-patient version. Those with a current or past diagnosis of major psychiatric disorders, dementia, or intellectual disability were excluded.

This study has been approved by the Ethical Committee at the Third Affiliated Hospital of Sun Yat-sen University ([2018]02-207-01).

To ensure accuracy of the diagnosis, all enrolled patients were followed up for at least six months, with a minimum of three follow-up visits. Patients who were hospitalized or had an existing diagnosis of bipolar disorder may have fewer follow-up visits.

Items noted for follow-up included:

1. Mood episodes.

These were assessed through direct observation by clinical physicians and inquiries with informants to determine the presence of hypomanic or mixed episodes during follow-up visits or hospitalization.

2. Prescriptions.

All enrolled patients initially diagnosed with depression were prescribed antidepressants without mood stabilizers (including lithium, anticonvulsants, and second-generation antipsychotics) whenever possible. Conversely, patients diagnosed with bipolar disorder were ideally treated with mood stabilizers without antidepressants. Sedative-hypnotic drugs were permitted as needed for all patients.

3. Treatment response.

During follow-up, response to antidepressants or mood stabilizers was determined based on the following criteria:

- Sustained response to antidepressants (with or without sedative-hypnotics) for six months supported a MDD diagnosis.
- Development of mania or mixed episodes after antidepressant treatment confirmed a BD diagnosis.
- Lack of response to antidepressants, but significant improvement after switching to mood stabilizers alone for six months, was also considered evidence of bipolar disorder.

Effective treatment was defined as meeting the following criteria:

- Continuous symptom improvement during follow-up.
- Clinical Global Impression (CGI) severity score of <4 at the last two consecutive visits.
- Treatment efficacy index score of ≤3 at each visit from the second visit onward.
- Partial or complete recovery of social functioning.
- No medication crossover (depression group without mood stabilizers; bipolar group without antidepressants).
- No manic switch.

Based on patients’ medical history, follow-up observations, and diagnostic treatment results, two attending or senior psychiatrists determined the final diagnosis for each patient according to the Diagnostic and Statistical Manual of Mental Disorders, Fourth Edition, Text Revision (DSM-IV-TR) criteria.

The clinical diagnostic interviews were conducted by researchers from the same clinical medical team. Before the project began, the two attending psychiatrists responsible for the final diagnosis underwent a one-month training program to ensure diagnostic consistency. Subsequently, they independently diagnosed 40 patients with bipolar disorder, achieving a diagnostic consistency rate of 0.975.

Participant demographics were collected through a self-designed questionnaire. Psychotic features were measured by evaluating whether the participants experienced any psychotic symptoms including hallucination, delusion or disorganized behavior during the past affective episodes. Physical comorbidities were confirmed by reviewing the patients’ previous medical history and electronic medical records stored in our hospital’s medical system. Mental comorbidities were diagnosed according to the DSM-IV-TR based on the subjects’ history of present illness and routine mental examination. Family history of mental disorders was assessed by asking the subjects or their accompanying relatives whether their first- or second-degree relatives had mental disorders of any kind. All assessments and interviews were conducted by trained psychiatrists from the study team.

Symptom severity of patients were evaluated with the 17- item Hamilton Depression Scale (HAMD-17) and the Young Mania Rating Scale (YMRS). To ensure quality and reliability of the evaluation, the same well-trained researcher (Zhaoyu Gan) was responsible for all assessments.

Personality traits of all participants were assessed using the Chinese version of the Big Five Inventory-44 (BFI-44), which has demonstrated reliability in Chinese samples (42). The questionnaire consists of 44 items rated on a 5-point Likert scale. Of these, 28 items are positively scored, while 16 items are negatively scored.

Statistical analyses were conducted using SPSS version 26 (44) and R Studio (45, 46). SPSS was used primarily for initial descriptive statistics, while main inferential analyses were conducted with R Studio. Negative-scored items were converted to positive scores prior to analysis. Kruskal-Wallis test was used to analyze continuous variables, including age and years of education, with the latter defined as the total number of years the participant spent in school or college. Categorical variables, such as marital status and history of traumatic events, were analyzed using Fisher’s exact test.

**Figure 1:**
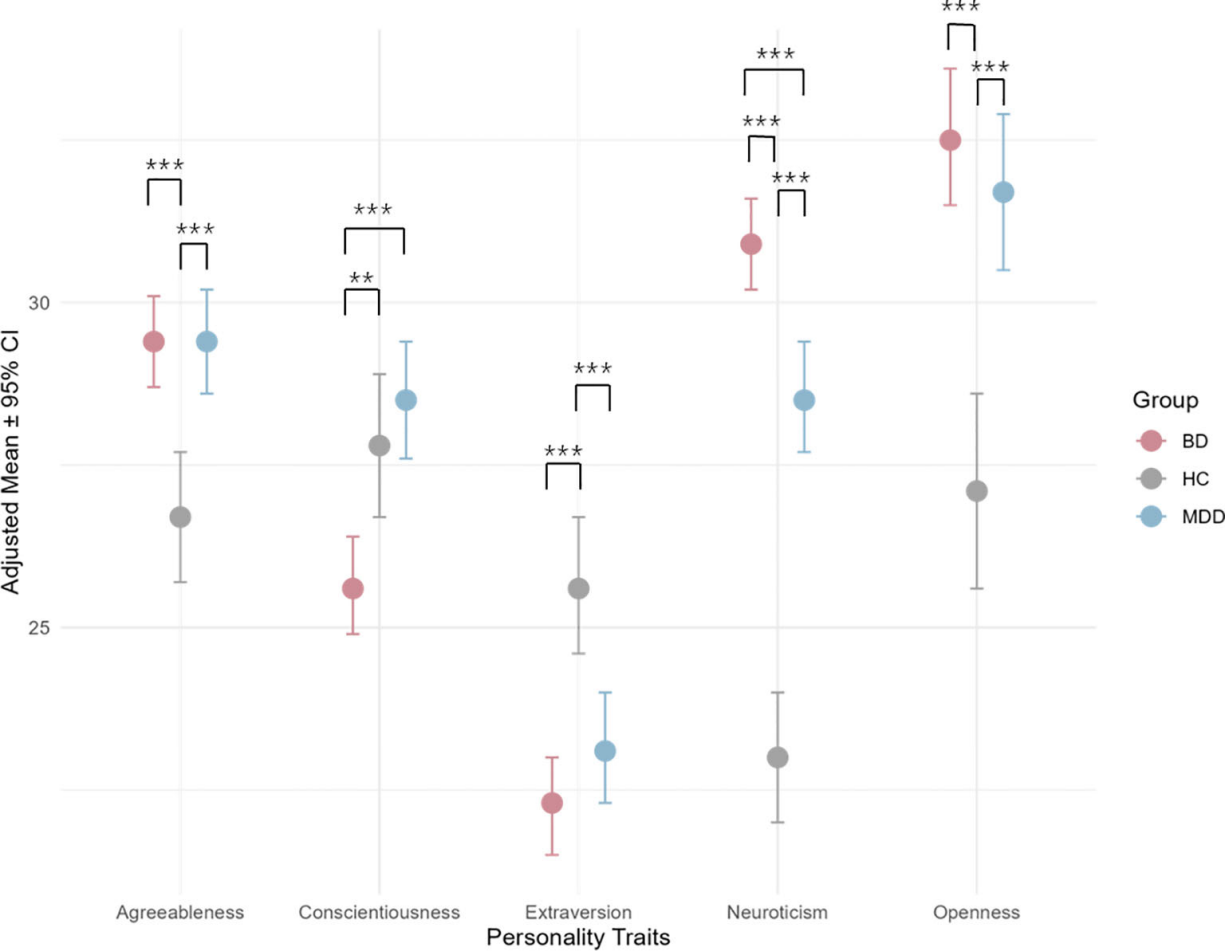
Adjusted mean scores of Big Five personality traits across groups. The asterisks denote levels of statistical significance in the post-hoc analysis: “***” for p<0.001, and “**” for p<0.01.

To examine differences in personality traits across diagnostic groups, an Analysis of Covariance (ANCOVA) was conducted in R Studio, adjusting for age and years of education. Of the 185 MDD patients, 73 had incomplete clinical data, leaving 112 for further analysis. Logistic regression analysis was performed to assess the association between clinical features and diagnosis (BD vs. MDD), with results reported as odds ratios (OR) and 95% confidence intervals (CI). The overall differences in personality traits between bipolar disorder (BD) and major depressive disorder (MDD) patients were examined using Multivariate Analysis of Variance (MANOVA). Once significance was confirmed using the Pillai test, univariate tests which controlled clinical features were performed, followed by pairwise comparisons for results with significance. All statistical significance levels were set at p < 0.05 (two-tailed), and numerical values were rounded to two decimal places when necessary. When running univariate regression for clinical features and BD, multicollinearity was detected. This was particular for first episode type (VIF = 284.15), psychiatric comorbidity (VIF = 284.05) and age (VIF = 9.50). The first two variables were removed eventually. In **Figure 1**, which showcases personality traits across groups, an extreme outlier in the Openness to Experience trait was removed for clearer visualization based on the 1.5*IQR criterion. However, all statistical analyses were conducted using the full dataset. Structural Equation Modeling (SEM) was performed following the regression analyses to further examine the relationships among personality traits, clinical features, and diagnostic outcomes. An iterative modeling approach was applied, in which only statistically significant predictors were retained. Final models were selected based on optimal fit indices, including Root Mean Square Error of Approximation (RMSEA), Comparative Fit Index (CFI), and other standard goodness-of-fit metrics.

## Results

### Subject demographics and clinical characteristics

Subject demographics and their clinical features are shown in **Table 1**. A total of 540 samples, including 252 individuals with Bipolar Disorder (BD), 185 with Major Depressive Disorder (MDD), and 103 healthy controls (HC) are included. The median age was 19.5 years for BD patients, 37 years for MDD patients, and 26 years for healthy controls. Years of education received varied across groups, with a median of 12 years in BD patients, 9 years in MDD patients, and 13 years in healthy controls. In terms of marital status, most BD patients were unmarried (83.73%), while a higher proportion of MDD patients were married (69.72%). Among BD patients, 50.78% were diagnosed with BD Type II (BD-II), 45.24% had mixed episodes, and 3.97% had BD Type I (BD-I). The median age of illness onset was 15 years in BD patients and 24 years in MDD patients. Baseline symptom severity was assessed using the HAMD and YMRS. HAMD scores were higher in MDD patients (median = 27) than BD patients (median = 22) and vice versa for YMRS scores – with a median score of 14 in BD patients compared to MDD patients (median = 4). Additional clinical features, including suicide history, physical comorbidities, and psychiatric comorbidities, are also summarized. For more details, please refer to **Supplementary Table 1**.

**Table 1:** Demographic characteristic and clinical features of study participants.

	BD (N=252)	MDD (N=185)	HC (N=103)	Test Statistic	p-value
Age (years), median [Q25,Q75]	19.5 [16.0; 25.0]	37.0[30.0; 44.5]	26.0 [19.5; 36.0]		<0.001
Years of Education, median [Q25,Q75]	12 [11.0; 15.0]	9 [6.0; 12.0]	13[12.00; 16.00]		<0.001
Marital status (N, %)					<0.001
Unmarried	211 (83.73%)	42 (22.70%)	56 (54.4%)		
Married	38 (15.08%)	129 (69.72%)	46 (44.7%)		
Divorced	3 (1.19%)	2 (1.08%)	0		
Other	0	12 (6.48%)	1 (0.9%)		
Bipolar disorder type					
Type I	10 (3.97%)				
Type II	128 (50.78%)				
Mixed	11 4(45.24%)				
Age of Illness Onset (years), median [Q25,Q75]	16.0 [14.0; 21.0]	24.0 [16.0; 34.0]			<0.001
HAMD score, median [Q25,Q75]	22 [17; 27]	25 [19; 29]			0.06
YMRS score, median [Q25,Q75]	14 [9; 20]	4 [0; 10]			<0.001
Traumatic experience					0.76
Yes	2 (0.79%)	3 (4.1%)			
No	250 (99.2%)	70 (95.9%)			
History of suicide attempt				*χ^2^ =* 2.48	0.12
Yes	30 (11.9%)	4 (5.5%)			
No	222 (88.10%)	69 (94.5)			
Physical comorbidities				*χ^2^ =* 0.54	0.46
Yes	56 (22.22%)	13 (17.8)			
No	196 (77.78%)	60 (82.2%)			
Psychiatric comorbidities				*χ^2^ =* 0.01	0.94
Yes	206 (81.75%)	60 (82.2%)			
No	46 (18.35%)	13 (17.8%)			
Hospitalization history					0.95
Yes	11 (4.36%)	3 (4.1%)			
No	241 (95.64%)	70 (95.9%)			

### Comparison of BFI-44 scores between BD, MDD and HC

**Figure 1** shows the adjusted means for personality traits across groups, controlling for age and years of education. Statistically significant pairwise comparisons are indicated with asterisks. BD patients had the highest adjusted mean (30.9) for neuroticism, followed by MDD patients (28.5) and HC (23.0). This pattern was also observed in Openness to experience. On the contrary, the HC group was highest for extraversion (25.6), followed by MDD (23.1) and BD (22.3). MDD patients had the highest adjusted mean for conscientiousness (28.5), followed by HC (27.8) and BD (25.6). MDD and BD patients share the same adjusted mean in agreeableness (29.4), which are higher than HC (26.7). Please see **Supplementary Table 2** for further details. In pairwise comparisons, BD patients had significantly higher adjusted means for neuroticism compared to both HC (estimate = -7.88, p < 0.001) and MDD (estimate = -2.36, p < 0.001). The comparison is also significant between MDD and HC, where MDD was notably higher (estimate = -5.52, p < 0.001). This order is also seen for Openness to Experience and Agreeableness. For the former, BD had the highest adjusted mean compared to both HC (estimate = -5.46, p < 0.001) and MDD (estimate = -0.84, p = 0.6125, not significant). MDD also had higher adjusted means than HC (estimate = -4.62, p < 0.001). In the latter, BD was significantly higher than HC (estimate = -2.66, p < 0.001) and MDD (estimate = 0.024, p = 0.995, not significant). MDD patients had significantly higher scores than HC (estimate = -2.68, p < 0.001). For extraversion, HC had significantly higher adjusted means than both MDD (estimate = 2.52, p < 0.001) and BD (estimate = 3.39, p < 0.001). This difference is not significant between MDD and BD (estimate = 0.86, p = 0.3486). BD patients had significantly lower adjusted means compared to both HC (estimate = 2.12, p = 0.0021) and MDD (estimate = 2.82, p < 0.001). The difference between HC and MDD was not statistically significant (estimate = -0.7, p = 0.6059). Further details are shown in **Supplementary Table 3**.

Pairwise comparisons, adjusting for clinical features, were conducted between MDD and BD (please see **Supplementary Table 4**). Among the five personality traits, only agreeableness showed significant difference between the two groups, with having MDD patients having a higher adjusted mean (32.1, 95% CI: [28.3, 36.0]) than BD (30.7, 95% CI: [27.1, 34.3]), while the rest were not statistically significant (p>0.05 for all comparisons).

### Comparison of BFI-44 scores between BD- I, BD II and BD-NOS

Adjusted means of the Big Five personality traits across BD subtypes is shown in **Supplementary Table 5**. BD-I had the highest adjusted mean in openness (32.8) and conscientiousness (28.9) compared to BD-II (30.2 and 27.3, respectively). Extraversion was similar across groups, with BD-I (22.7) and BD-II (22.6) being slightly higher than BD-NOS (21.0). Agreeableness and neuroticism barely differed between groups.

**Table 2** shows the *post-hoc* analysis of BFI traits across BD subtypes. Pairwise comparisons of adjusted means revealed significant differences in openness to experience, with BD-I scoring higher than BD-II (2.78, p = 0.02). Conscientiousness was also significantly higher in BD-I compared to BD-NOS (3.51, p = 0.03). No significant differences were observed for extraversion, agreeableness, or neuroticism (p > 0.05).

**Table 2:** *Post-hoc* analysis of BFI between BD-I, BDII and BD-NOS patients.

Personality trait	Contrast	Estimate	SE	df	t-ratio	Lower CI	Upper CI	p-value
Openness to Experience	BD-I-BD-II	2.78	1.04	232	2.67	0.32	5.23	**0.02**
	BD-I-BD-NOS	1.77	1.52	232	1.17	-1.8	5.35	0.47
	BD-II-BD-NOS	-1	1.5	232	-0.67	-4.53	2.53	0.78
Conscientiousness	BD-I-BD-II	1.98	0.96	232	2.06	-0.29	4.25	0.1
	BD-I-BD-NOS	3.51	1.4	232	2.5	0.2	6.82	**0.03**
	BD-II-BD-NOS	1.53	1.38	232	1.11	-1.74	4.8	0.51
Extraversion	BD-I-BD-II	0.24	0.8	232	0.3	-1.66	2.14	0.95
	BD-I-BD-NOS	1.83	1.17	232	1.57	-0.93	4.6	0.26
	BD-II-BD-NOS	1.5	1.16	232	1.38	-1.13	4.32	0.35
Agreeableness	BD-I-BD-II	-0.23	0.82	232	-0.28	-2.17	1.71	0.96
	BD-I-BD-NOS	0.81	1.2	232	0.67	-2.02	3.63	0.78
	BD-II-BD-NOS	1.03	1.18	232	0.87	-1.76	3.83	0.66
Neuroticism	BD-I-BD-II	-0.24	0.81	232	-0.3	-2.15	1.66	-0.95
	BD-I-BD-NOS	-1.17	1.18	232	-0.99	-3.94	1.61	-0.58
	BD-II-BD-NOS	-0.93	1.16	232	-0.8	-3.67	1.82	-0.71

SE, standard error; df, degrees of freedom; CI, confidence interval.

Bolded p-values indicate statistical significance at p < 0.05.

### Association between personality traits and clinical features

Multiple linear regression was conducted to evaluate the association between Big Five personality traits and clinical features of mood disorders. Among the significant findings, Openness to Experience was negatively associated with HAMD scores (β = -0.15, p < 0.001), while extraversion was marginally associated with cumulative depressed days negatively (β = -0.12, p = 0.05). Neuroticism showed high association with HAMD scores (β = 0.26, p < 0.001) and a history of suicide attempt (β = 0.23, p = 0.16), though the latter is not statistically significant. High agreeableness is associated with the number of hospitalizations (β = 0.10, p = 0.04), and less depressed days (β = -0.11, p = 0.04). BD patients show lower agreeableness compared to MDD (β = -0.14, p = 0.04). For details, please see **Supplementary Table 6**.

Regression analyses identified several clinical features that were associated with personality traits in bipolar disorder (shown in **Supplementary Table 7**). Higher HAMD scores were associated with lower openness (-0.23, p = 0.01), lower conscientiousness (-0.14, p = 0.04), lower extraversion (-0.27, p < 0.001), and higher neuroticism (0.38, p < 0.001). Higher educational level linked to higher conscientiousness (0.24, p = 0.01) and agreeableness (0.13, p = 0.05). Physical comorbidities were positively associated with openness (0.19, p = 0.01). Number of past hospitalizations were correlated to higher agreeableness (0.29, p = 0.02). No other significant associations were observed (p > 0.05).

Logistic regression was conducted to examine the association between clinical features and different mood disorder diagnoses (shown in **Supplementary Table 8**). Significant predictors of BD diagnosis included earlier illness onset (OR = 0.85, p = 0.001), later age at first psychiatric appointment (OR = 1.1, p = 0.01), and having psychiatric comorbidities (OR = 2.15, p = 0.002). Hospitalization history was also associated with increased BD likelihood (OR = 7.56, p = 0.05), though its significance was borderline. Years of education received also showed a weak negative association with BD (OR = 0.90, p = 0.05).

### Structural equation modeling

**Figure 2:**
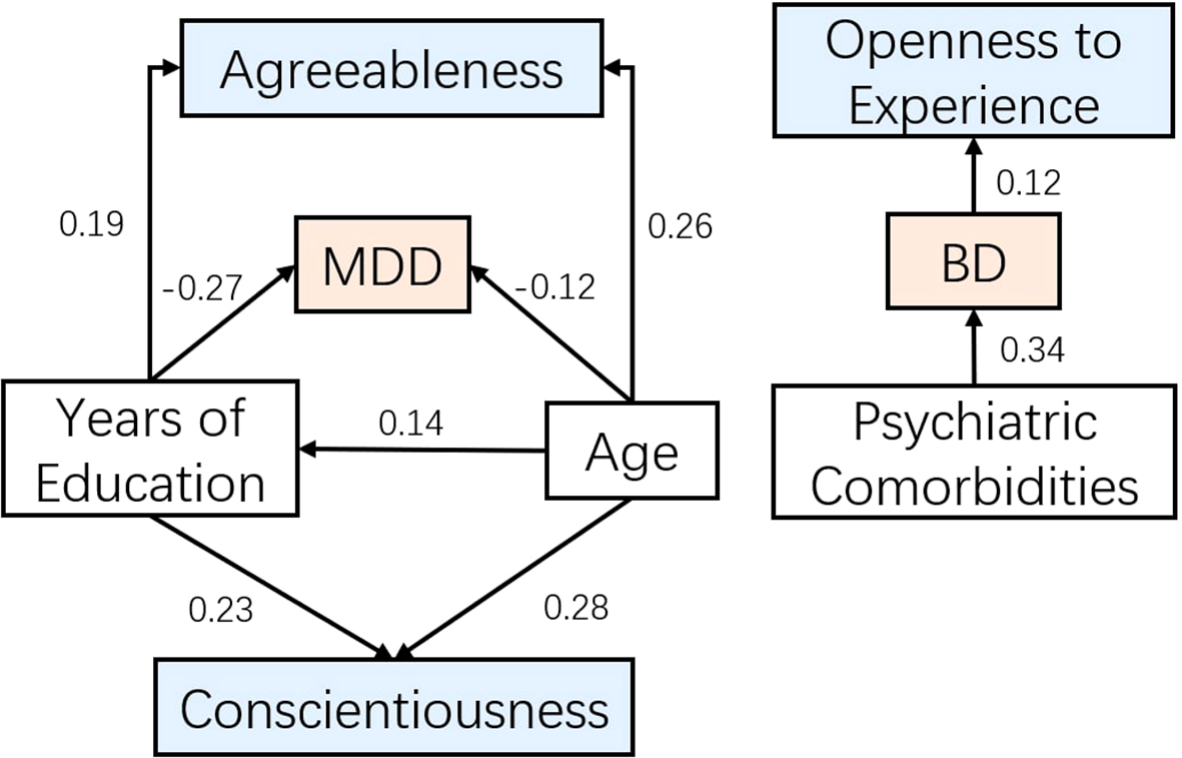
Structural equation model depicting the associations among personality traits, demographic variables, and diagnosis.

To build upon and further validate our regression findings, a structural equation model (SEM) was conducted to examine the associations among Big Five personality traits, clinical features, demographic factors, and diagnosis (**Figure 2**).

Despite the limited global fit, several pathways within the model were statistically significant and clinically meaningful. Residual variances for all endogenous variables were significant (p < 0.001), indicating that unexplained variance remained for each outcome.

Age and educational attainment were positively associated with higher levels of Conscientiousness (β = 0.284, p < 0.001; β = 0.227, p < 0.001) and Agreeableness (β = 0.255, p < 0.001; β = 0.189, p < 0.001). Depression severity (HAMD scores) was positively associated with Neuroticism (β = 0.199, p < 0.001).

Regarding diagnostic prediction, higher Openness (β = 0.121, p < 0.001), greater psychiatric comorbidity (β = 0.343, p = 0.031), and younger age (β = -0.265, p < 0.001) were significantly associated with an increased likelihood of a BD diagnosis relative to MDD. Conscientiousness, Agreeableness, Extraversion, Neuroticism, and educational attainment were not significantly associated with diagnostic status after accounting for other predictors.

## Discussion

Neuroticism, extraversion and conscientiousness differed significantly across three groups. Neuroticism in BD and MDD were higher than HC, with BD being the highest, confirming its relationship with psychiatric disorders (9, 47, 48), regardless of illness state. Some studies suggest that extraversion can serve as a predictor for diagnosis between BD and MDD (11). In our study, both illness groups exhibited significantly lower extraversion than the control group, while the BD group scored slightly lower than MDD, though the difference was not statistically significant. This is consistent with most studies (12, 18, 49–51), suggesting that low extraversion (introversion) is notable for distinguishing patients with mood disorders, but lacks reliability when differentiating between bipolar and unipolar depression. Interestingly, agreeableness did not significantly differ between MDD and BD in the *post hoc* analysis but became significant after adjusting for clinical features, with MDD (32.1) scoring higher than BD (30.7). This made agreeableness the only distinguishable trait between MDD and BD in our study. This may be explained by pervious findings where mania is found to be associated with low agreeableness (11). Given that agreeableness is often linked to interpersonal functioning, emotional regulation, and impulse control, its lower levels in BD compared to MDD may reflect the greater social and behavioral dysregulation associated with bipolar disorder, particularly in manic states. However, not all prior studies have found this association, with some research in Western populations reporting no significant difference in agreeableness between MDD and BD (52). This discrepancy may be influenced by cultural norms, as Chinese women tend to score higher on agreeableness (53), due to the societal emphasis on social harmony and collectivism, which may minimize trait differences across clinical groups.

Other personality traits no longer exhibited significant differences after adjustment for clinical features, suggesting that they are largely driven by clinical symptoms rather than stable personality differences. This finding reinforces the idea that agreeableness may be a more stable trait independent of symptom fluctuations, distinguishing BD from MDD, which is consistent with previous studies suggesting that agreeableness remains stable throughout the illness course (47, 48).

When examining the relationship between clinical features and personality traits, depression severity (HAMD scores) showed multiple associations. To minimize confounding, we re-ran the regression model without HAMD as a covariate, but this did not meaningfully alter the results, suggesting that the observed effects were not primarily driven by depression severity. Depression severity was highly associated with neuroticism, which is consistent with prior studies (11), and negatively correlated with conscientiousness, openness to experience, and extraversion. Interestingly, these traits correlated specifically with HAMD scores but not with other indicators of illness severity (e.g., suicidality, number of depressive episodes). This suggests that personality traits are more strongly linked to the intensity of current depressive symptoms rather than the cumulative burden of illness over time.

A novel finding was the association between physical comorbidities and openness to experience, which may indicate greater health awareness among individuals high in openness or a greater tendency to disclose health information. Additionally, years of education and age were positively associated with openness to experience and conscientiousness, aligning with previous research that suggests extraversion remains stable over time, while openness declines and conscientiousness increases (23, 24, 39). However, these patterns may differ in psychiatric populations, possibly due to selective attrition, where individuals with higher openness are more likely to remain engaged in mental health care and research participation.

In terms of clinical relevance, early illness onset, psychiatric comorbidities, and hospitalization history were all indicative of a BD diagnosis. Later age at first psychiatric appointment was also associated with BD, suggesting that patients tend to experience mood symptoms earlier but take longer to seek help. This delay may reflect misdiagnosis as unipolar depression and may contribute to the increased likelihood of hospitalization.

Within BD patients, neuroticism did not differ significantly, indicating that it is a shared trait across mood disorders, differentiating psychiatric patients from healthy controls but not within diagnostic subtypes. BD-I patients exhibited higher openness and conscientiousness compared to BD-II and BD-NOS, a pattern that aligns with existing literature (22). Low openness has been associated with depression and anxiety (51), which fits with BD-II patients experiencing more depressive than manic symptoms, while BD-I patients exhibit greater risk-taking tendencies. Similarly, higher conscientiousness in BD-I could reflect over-planning and increased goal-directed activity associated with manic symptoms.

Across both BD and MDD, HAMD scores were associated with lower openness and extraversion, while strongly correlating with neuroticism, reinforcing neuroticism as a core marker of psychiatric illness. Within the BD group, high agreeableness was associated with a history of hospitalization, possibly reflecting better adherence to treatment among more agreeable individuals. This finding suggests an avenue for future research into how personality traits influence psychiatric care engagement.

Finally, conscientiousness was negatively correlated with HAMD scores within BD, but this association disappeared when analyzed across both BD and MDD. This finding is consistent with some studies (18, 51) indicating that BD patients exhibit greater variability in motivation and goal-directed behavior depending on mood state, whereas MDD is characterized by persistently low energy and diminished motivation. This suggests that conscientiousness may be more reactive to acute depressive episodes in BD, while remaining consistently low in MDD regardless of symptom severity.

In addition to regression analyses, the SEM approach offered a more integrative framework, corroborating and extending the regression findings. Specifically, it confirmed that lower conscientiousness and agreeableness, higher openness, and the presence of psychiatric comorbidities differentially predicted diagnostic outcomes. Overall, the SEM results complemented the regression analyses by reinforcing the differential contributions of personality traits, clinical features, and demographic variables to the likelihood of a BD versus MDD diagnosis—highlighting the distinct roles of higher openness, psychiatric comorbidity, and younger age in identifying BD.

However, the overall model fit indices were suboptimal, suggesting that while the hypothesized pathways were theoretically and clinically meaningful, the model structure requires further refinement. Future studies with larger samples and potentially alternative modeling approaches are needed to validate and extend these findings.

In summary, our study highlights the role of personality traits in BD and MDD, with neuroticism as a key marker of psychiatric illness and agreeableness distinguishing BD from MDD after adjustment. While conscientiousness and openness were influenced by clinical and demographic factors, extraversion and openness were more closely linked to current depressive severity than long-term illness burden. These results emphasize the importance of personality traits in understanding illness course and treatment engagement, supporting further research into their role in personalized psychiatry and intervention strategies.

### Strength and limitations

Given the subtle trait differences observed across groups, ensuring methodological rigor is essential for drawing valid conclusions. One of the key strengths of this study is its large sample size, which enhances statistical power and generalizability. Additionally, all patients were recruited from the same hospital, ensuring uniformity in diagnostic procedures, clinical assessments, and treatment protocols, thereby minimizing variability introduced by differences in clinical settings. This single-site recruitment approach helps control for institutional and regional differences in mental health care, which can be a major source of confounding in multi-center studies. Furthermore, our results were adjusted for age and education, which are known to influence personality traits. The unadjusted means followed a similar trend, though some group differences were less pronounced prior to adjustment. This highlights the importance of controlling demographic factors when examining personality traits in clinical populations, ensuring that observed effects are not merely a reflection of sample differences in sociodemographic characteristics.

However, this study has several limitations. The sample consisted only of Han Chinese women, which limits generalizability to other genders and ethnicities. Additionally, participants were recruited from a single hospital, which may not represent broader psychiatric populations. The reliance on treatment response for diagnostic confirmation introduces potential misclassification, as some bipolar disorder cases may not have experienced mood switches during follow-up. Family history of mental illness was based on self-reports, which are prone to recall bias. Medication effects on personality and symptom severity were not fully controlled for, potentially influencing observed differences between groups. Finally, all clinical severity assessments were conducted by a single researcher, introducing potential rater bias. Furthermore, while follow-up visits helped refine diagnoses, the assessment of personality traits was cross-sectional, meaning transient mood states could have influenced responses, limiting conclusions about stable personality differences. clinical data were missing for a substantial proportion of MDD patients, particularly for symptom severity, comorbidities, and medication history, which may have introduced bias in group comparisons. The use of a single personality measure may also not fully capture relevant trait dimensions. Although previous research has shown that the Chinese version of the BFI-44 has significantly lower reliability than the English version, especially in the Extraversion and Openness subscales (54), a recent research has shown promising reliability (55). Due to the requirement of a six-month follow-up period to confirm diagnoses in patient groups, a non-parallel enrollment process for healthy controls was inevitable. However, this approach ensured that all controls included in the analysis were age- and sex-matched to the cases. Despite this effort, sensitivity analysis revealed demographic differences between groups, and this was addressed by subsequent subgroup analyses and regression models to minimize the potential confounding effects of these demographic variables on the results. Finally, the SEM exhibited poor global fit indices, limiting the interpretability of the model as a whole. Nonetheless, individual path estimates remained statistically meaningful and were reported for exploratory purposes.

## Data Availability

The raw data supporting the conclusions of this article will be made available by the authors, without undue reservation.
