# Supplementary material for: Unraveling personality in mood disorders: the role of big five personality traits in Han Chinese women with bipolar and unipolar depression
Source: Front Psychiatry. 2025 Jun 13;16:1596956. doi: 10.3389/fpsyt.2025.1596956 (PMC12202330; doi:10.3389/fpsyt.2025.1596956)
Supplement: Supplementary file 1 [file Table1.docx]

**Supplementary Table 1: Clinical Features of Study Participants (Continued)**

|  | **BD (N=252)** | **MDD (N=185)** | **p value** |
| --- | --- | --- | --- |
| **Number of physical comorbidities** |  |  | 0.46 (*χ2*=0.54) |
| 0 | 196 (77.78%) | 60 (82.2%) |  |
| 1 | 53 (21.03%) | 13 (17.8%) |  |
| 2 | 3 (1.19%) | 0 |  |
| **Number of psychiatric comorbidities** |  |  | 0.935 (*χ2*=0.007) |
| 0 | 46 (18.35%) | 13 (17.8%) |  |
| 1 | 137 (54.37%) | 60 (82.2%) |  |
| 2 | 9 (3.57%) | 0 |  |
| 3 | 60 (23.8%) | 0 |  |
| **Number of given births** |  |  | **0.002** (*χ²*=16.5) |
| 0 | 207 (82.14%) | 47 (64.4%) |  |
| 1 | 15 (5.95%) | 8 (11.0%) |  |
| 2 | 16 (6.35%) | 15 (20.5%) |  |
| 3 | 4 (1.58%) | 1 (1.4%) |  |
| ≥4 | 10 (3.97%) | 2 (2.8%) |  |
| **Postpartum depression** |  |  | 0.484 (*χ²*=0.49) |
| Yes | 18/45 (39.56%) | 7/26 (26.9%) |  |
| No | 27/45 (60.44) | 19/26 (73.1%) |  |
| **Family history of psychiatric illness** |  |  | 0.763 |
| Yes | 32 (12.70%) | 8 (11.0%) |  |
| No | 220 (87.30%) | 65 (89%) |  |
| **Onset episode type** |  |  |  |
| Depression | 138 (54.76%) |  |  |
| (Hypo)Mania | 9 (3.57%) |  |  |
| Mixed | 60 (23.80%) |  |  |
| Unspecified | 45 (17.86%) |  |  |
| **Age at 1^st^ appointment** (years), median [Q25,Q75] | 19.0 [16.0; 24.0] | 26.0 [20.0;33.0] | **<0.001** |
| **Age at medication** (years), median [Q25,Q75] | 21[16.00; 25.00] | 25 [18.50;26.00] | **0.019** |
| **Age at 1^st^ hospitalization** (years) |  |  | 0.7 |
| Never hospitalized | 238 (94.45%) | 69 (94.5%) |  |
| <12 years | 1 (0.40%) | 1 (1.40%) |  |
| 13~24 years | 7 (2.78%) | 1 (1.40%) |  |
| 25~30 years | 3 (1.19%) | 0 (0.00%) |  |
| >30 years | 3 (1.19%) | 2 (2.8%) |  |
| **Hospitalization History** |  |  | 0.952 |
| Yes | 11 (4.36%) | 3 (4.1%) |  |
| No | 241 (95.64%) | 70 (95.9%) |  |
| **Number of hospitalizations** |  |  | 0.983 |
| 0 | 241 (95.64%) | 70 (95.9%) |  |
| 1 | 5 (1.98%) | 3 (4.1%) |  |
| 2 | 3 (1.19%) | 0 (0.00%) |  |
| 3 | 3 (1.19%) | 0 (0.00%) |  |
| **Number of depressive episodes**, median [Q25,Q75] | 2 [0.00;3.00] | 2 [1.00;3.00] | 1.000 |
| **Cumulative depressed days**, median [Q25,Q75] | 40 [9.25; 120.00] | 120 [60.00;300.00] | **<0.001** |
| **Number of (hypo)manic episodes**, median [Q25,Q75] | 1 [0.00; 2.00] |  | 0.254 |
| Median |  |  |  |
| [Q25,Q75] |  |  |  |
| **Cumulative (hypo)manic days**, median [Q25,Q75] | 14 [1.25; 90.00] |  |  |
| **Whether mixed** |  |  |  |
| Yes | 114 (45.24%) |  |  |
| No | 138 (54.76%) |  |  |
| **Number of mixed episodes** |  |  |  |
| 0 | 138 (54.76%) |  |  |
| 1 | 100 (39.68%) |  |  |
| 2 | 10 (3.97%) |  |  |
| 3 | 3 (1.19%) |  |  |
| ≥4 | 1 (0.40%) |  |  |
| **Cumulative mixed days**, median [Q25,Q75] | 0 [0.00; 180.00] |  |  |

Note: HC = Healthy controls, MDD = major depressive disorder, BD = bipolar disorder. A total of 34 participants (13.49%) with BD and 7 participants (9.6%) with MDD were never medicated.

Bolded p-values indicate statistical significance at p < .05.

**Supplementary Table 2: Big Five Personality Scores Across Groups**

| **Personality Trait** | **Group** | **Estimated Means** | **Standard Error** | **Degree of Freedom** | **Lower CI** | **Upper CI** |
| --- | --- | --- | --- | --- | --- | --- |
| **Neuroticism** | **HC** | 23.0 | 0.52 | 535 | 22.0 | 24.0 |
|  | **MDD** | 28.5 | 0.43 | 535 | 27.7 | 29.4 |
|  | **BD** | 30.9 | 0.35 | 535 | 30.2 | 31.6 |
| **Extraversion** | **HC** | 25.6 | 0.53 | 535 | 24.6 | 26.7 |
|  | **MDD** | 23.1 | 0.44 | 535 | 22.3 | 24.0 |
|  | **BD** | 22.3 | 0.36 | 535 | 21.5 | 23.0 |
| **Openness to Experience** | **HC** | 27.1 | 0.76 | 535 | 25.6 | 28.6 |
|  | **MDD** | 31.7 | 0.63 | 535 | 30.5 | 32.9 |
|  | **BD** | 32.5 | 0.52 | 535 | 31.5 | 33.6 |
| **Agreeableness** | **HC** | 26.7 | 0.52 | 535 | 25.7 | 27.7 |
|  | **MDD** | 29.4 | 0.43 | 535 | 28.6 | 30.2 |
|  | **BD** | 29.4 | 0.35 | 535 | 28.7 | 30.1 |
| **Conscientiousness** | **HC** | 27.8 | 0.56 | 535 | 26.7 | 28.9 |
|  | **MDD** | 28.5 | 0.46 | 535 | 27.6 | 29.4 |
|  | **BD** | 25.6 | 0.39 | 535 | 24.9 | 26.4 |

Note: HC = Healthy controls, MDD = major depressive disorder, BD = bipolar disorder. CI = confidence interval.

**Supplementary Table 3: Post-hoc Analysis of Big Five Personality Scores Across Groups**

| **Personality Trait** | **Contrast** | **Adjusted Means** | **Standard Error** | **t-ratio** | **Degree of Freedom** | **Lower CI** | **Upper CI** | **p-value** |
| --- | --- | --- | --- | --- | --- | --- | --- | --- |
| **Neuroticism** | **HC-MDD** | -5.52 | 0.68 | -8.17 | 535 | -7.11 | -3.93 | **<0.001** |
|  | **HC-BD** | -7.88 | 0.63 | -12.53 | 535 | -9.36 | -6.40 | **<0.001** |
|  | **MDD-BD** | -2.36 | 0.60 | -3.93 | 535 | -3.78 | -0.95 | **<0.001** |
| **Extraversion** | **HC-MDD** | 2.52 | 0.7 | 3.62 | 535 | 0.88 | 4.15 | **<0.001** |
|  | **HC-BD** | 3.39 | 0.65 | 5.21 | 535 | 1.85 | 4.90 | **<0.001** |
|  | **MDD-BD** | 0.86 | 0.62 | 1.39 | 535 | -0.6 | 2.31 | 0.3486 |
| **Openness to Experience** | **HC-MDD** | -4.62 | 0.99 | -4.65 | 535 | -6.96 | -2.29 | **<0.001** |
|  | **HC-BD** | -5.46 | 0.93 | -5.9 | 535 | -7.64 | -3.28 | **<0.001** |
|  | **MDD-BD** | -0.84 | 0.89 | -0.94 | 535 | -2.92 | 1.24 | 0.6125 |
| **Agreeableness** | **HC-MDD** | -2.68 | 0.68 | -3.97 | 535 | -4.27 | -1.10 | **<0.001** |
|  | **HC-BD** | -2.66 | 0.63 | -4.23 | 535 | -4.13 | -1.18 | **<0.001** |
|  | **MDD-BD** | 0.02 | 0.6 | 0.04 | 535 | -1.39 | 1.44 | 0.9991 |
| **Conscientiousness** | **HC-MDD** | -0.70 | 0.74 | -0.95 | 535 | -2.15 | 0.76 | 0.6095 |
|  | **HC-BD** | 2.12 | 0.69 | 3.08 | 535 | 0.77 | 3.46 | **<0.01** |
|  | **MDD-BD** | 2.82 | 0.66 | 4.29 | 535 | 1.51 | 4.13 | **<0.001** |

Note: HC = Healthy controls, MDD = major depressive disorder, BD = bipolar disorder. CI = confidence interval. Bolded p-values indicate statistical significance at p < 0.05.

**Supplementary Table 4: Post-hoc Analysis of Big Five Personality Scores Between BD and MDD**

| **Personality Trait** | **Group** | **Adjusted Means (SE)** | **95% CI [Upper; Lower]** | **Contrast Estimate (SE)** | **t-ratio** | **p-value** |  |
| --- | --- | --- | --- | --- | --- | --- | --- |
|  |  |  |  |  |  |  |  |
|  |  |  |  |  |  |  |  |
| **Openness to Experience** | **MDD** | 31.6 (2.06) | [27.5; 35.6] | -0.75 (1.30) | -0.58 | 0.56 |  |
|  | **BD** | 32.3 (1.99) | [28.4; 36.3] |  |  |  |  |
| **Conscientiousness** | **MDD** | 28.1 (1.49) | [25.1; 31.0] | 0.96 (0.10) | 0.96 | 0.34 |  |
|  | **BD** | 27.1 (1.28) | [24.6; 29.6] |  |  |  |  |
| **Extraversion** | **MDD** | 23.2 (1.33) | [20.6; 25.8] | 0.11 (0.80) | 0.13 | 0.89 |  |
|  | **BD** | 23.1 (1.24) | [20.6; 25.5] |  |  |  |  |
| **Agreeableness** | **MDD** | 32.1 (1.95) | [28.3; 36.0] | 1.46 (0.72) | 2.03 | **0.04** |  |
|  | **BD** | 30.7 (1.81) | [27.1; 34.3] |  |  |  |  |
| **Neuroticism** | **MDD** | 29.5 (1.57) | [26.4; 32.6] | -0.61 (0.80) | -0.77 | 0.44 |  |
|  | **BD** | 30.1 (1.47) | [27.2; 33.0] |  |  |  |  |

Note: CI = confidence interval. Bolded p-values indicate statistical significance at p < 0.05.

Results were adjusted for adjusted for age, marital status, years of education, age at illness onset, Hamilton Depression Rating Scale score, age at first hospitalization, number of depressive episodes, cumulative depressed days, medication status, history of trauma, family history of psychiatric illness, number of physical comorbidities, number of psychiatric comorbidities, history of suicide attempts, age at first psychiatric appointment, age at first medication use, and number of psychiatric hospitalizations.

**Supplementary Table 5: Post-hoc Analysis of Big Five Personality Scores Between BD and MDD**

| **Personality Trait** | **Group** | **Estimated Means** | **Standard Error** | **Degree of Freedom** | **Lower CI** | **Upper CI** |
| --- | --- | --- | --- | --- | --- | --- |
| **Openness to Experience** | **Type I** | 32.8 | 2.69 | 230 | 27.5 | 38.1 |
|  | **Type II** | 30.2 | 2.73 | 230 | 24.8 | 35.6 |
|  | **NOS** | 31.7 | 3.02 | 230 | 25.7 | 37.6 |
| **Conscientiousness** | **Type I** | 28.9 | 2.49 | 230 | 24.0 | 33.8 |
|  | **Type II** | 27.3 | 2.53 | 230 | 22.3 | 32.3 |
|  | **NOS** | 25.9 | 2.80 | 230 | 20.4 | 31.4 |
| **Extraversion** | **Type I** | 22.7 | 2.09 | 230 | 18.6 | 26.8 |
|  | **Type II** | 22.6 | 2.12 | 230 | 18.5 | 26.8 |
|  | **NOS** | 21.0 | 2.35 | 230 | 16.4 | 25.6 |
| **Agreeableness** | **Type I** | 30.3 | 2.15 | 230 | 26.0 | 34.5 |
|  | **Type II** | 30.5 | 2.18 | 230 | 26.2 | 34.8 |
|  | **NOS** | 29.5 | 2.42 | 230 | 24.8 | 34.3 |
| **Neuroticism** | **Type I** | 29.9 | 2.10 | 230 | 25.8 | 34.0 |
|  | **Type II** | 30.1 | 2.13 | 230 | 25.9 | 34.3 |
|  | **NOS** | 30.7 | 2.36 | 230 | 26.1 | 35.4 |

Note: HC = Healthy controls, MDD = major depressive disorder, BD = bipolar disorder. NOS = not otherwise specified. CI = confidence interval. Bolded p-values indicate statistical significance at p < 0.05.

**Supplementary Table 6: Associations Between Big Five Personality Traits and Clinical Features in Mood Disorders**

| **Trait** | **Predictor** | **Standardized β** | **Standard Error** | **t value** | **p value** | **95% CI (Lower, Upper)** |
| --- | --- | --- | --- | --- | --- | --- |
| **Openness to Experience** | (Intercept) | -0.06 | 0.10 | -0.55 | 0.58 | -0.26, 0.14 |
|  | Age | 0.12 | 0.19 | 0.63 | 0.53 | -0.29, 0.50 |
|  | Marital Status | 0.10 | 0.18 | 0.56 | 0.57 | -0.25, 0.45 |
|  | Years of Education | 0.30 | 0.09 | 3.41 | 0.00 | 0.13, 0.47 |
|  | Diagnosis | 0.05 | 0.09 | 0.58 | 0.56 | -0.12, 0.22 |
|  | Age at Illness Onset | -0.07 | 0.15 | -0.44 | 0.66 | -0.36, 0.23 |
|  | HAMD Score | -0.21 | 0.05 | -4.24 | **<0.001** | -0.3, -0.11 |
|  | Age at First Hospitalization | -0.08 | 0.06 | -1.33 | 0.19 | -0.20, 0.04 |
|  | Number of Depressive Episodes | 0.07 | 0.05 | 1.30 | 0.19 | -0.03, 0.17 |
|  | Cumulative Depressed Days | -0.07 | 0.06 | -1.21 | 0.23 | -0.19, 0.05 |
|  | Whether Medicated | 0.00 | 0.09 | -0.03 | 0.97 | -0.18, 0.17 |
|  | Trauma History | 0.00 | 0.05 | 0.06 | 0.95 | -0.10, 0.11 |
|  | Family History of Psychiatric Disorsder | 0.04 | 0.06 | 0.63 | 0.53 | -0.08, 0.15 |
|  | Physical Comorbidiy | 0.14 | 0.06 | 2.35 | 0.02 | 0.02, 0.26 |
|  | Psychiatric Comorbidity | -0.03 | 0.06 | -0.48 | 0.63 | -0.13, 0.08 |
|  | Suicide Attempt | -0.04 | 0.05 | -0.87 | 0.38 | -0.13, 0.05 |
|  | Age at First Appointment | -0.24 | 0.16 | -1.45 | 0.15 | -0.56, 0.08 |
|  | Age at Initial Medication | -0.02 | 0.09 | -0.26 | 0.80 | -0.21, 0.16 |
|  | Number of Hospitalizations | -0.02 | 0.07 | -0.28 | 0.78 | -0.16, 0.12 |
| **Conscientiousness** | (Intercept) | -0.08 | 0.08 | -0.97 | 0.33 | -0.24, 0.08 |
|  | Age | 0.39 | 0.18 | 2.18 | **0.03** | 0.04, 0.74 |
|  | Marital Status | 0.13 | 0.14 | 0.90 | 0.37 | -0.15, 0.41 |
|  | Years of Education | 0.22 | 0.09 | 2.52 | **0.01** | 0.05, 0.38 |
|  | Diagnosis | -0.07 | 0.08 | -0.96 | 0.34 | -0.22, 0.08 |
|  | Age at Illness Onset | 0.14 | 0.14 | 0.99 | 0.32 | -0.14, 0.41 |
|  | HAMD Score | -0.13 | 0.06 | -2.21 | **0.03** | -0.25, -0.02 |
|  | Age at First Hospitalization | 0.06 | 0.11 | 0.55 | 0.58 | -0.15, 0.26 |
|  | Number of Depressive Episodes | 0.04 | 0.05 | 0.74 | 0.46 | -0.06, 0.13 |
|  | Cumulative Depressed Days | -0.02 | 0.07 | -0.29 | 0.77 | -0.17, 0.13 |
|  | Whether Medicated | -0.04 | 0.08 | -0.53 | 0.60 | -0.21, 0.12 |
|  | Trauma History | 0.05 | 0.03 | 1.50 | 0.14 | -0.02, 0.12 |
|  | Family History of Psychiatric Disorsder | 0.03 | 0.06 | 0.50 | 0.61 | -0.08, 0.14 |
|  | Physical Comorbidiy | 0.06 | 0.06 | 1.04 | 0.30 | -0.05, 0.18 |
|  | Psychiatric Comorbidity | -0.08 | 0.05 | -1.55 | 0.12 | -0.18, 0.02 |
|  | Suicide Attempt | 0.05 | 0.06 | 0.81 | 0.42 | -0.07, 0.17 |
|  | Age at First Appointment | -0.24 | 0.13 | -1.79 | 0.07 | -0.49, 0.02 |
|  | Age at Initial Medication | 0.05 | 0.09 | 0.61 | 0.54 | -0.12, 0.22 |
|  | Number of Hospitalizations | -0.04 | 0.12 | -0.30 | 0.77 | -0.28, 0.21 |
| **Extraversion** | (Intercept) | -0.08 | 0.09 | -0.89 | 0.38 | -0.25, 0.10 |
|  | Age | -0.03 | 0.19 | -0.18 | 0.85 | -0.40, 0.33 |
|  | Marital Status | 0.21 | 0.14 | 1.46 | 0.15 | -0.07, 0.48 |
|  | Years of Education | -0.07 | 0.09 | -0.80 | 0.42 | -0.26, 0.11 |
|  | Diagnosis | -0.01 | 0.07 | -0.13 | 0.89 | -0.15, 0.13 |
|  | Age at Illness Onset | 0.18 | 0.14 | 1.23 | 0.22 | -0.10, 0.46 |
|  | HAMD Score | -0.22 | 0.06 | -3.69 | 0.00 | -0.34, -0.10 |
|  | Age at First Hospitalization | 0.09 | 0.11 | 0.76 | 0.45 | -0.14, 0.31 |
|  | Number of Depressive Episodes | 0.02 | 0.06 | 0.34 | 0.73 | -0.10, 0.14 |
|  | Cumulative Depressed Days | -0.12 | 0.06 | -1.99 | 0.05 | -0.23, -0.01 |
|  | Whether Medicated | 0.05 | 0.08 | 0.59 | 0.56 | -0.11, 0.20 |
|  | Trauma History | 0.06 | 0.05 | 1.28 | 0.20 | -0.03, 0.15 |
|  | Family History of Psychiatric Disorsder | 0.05 | 0.06 | 0.86 | 0.39 | -0.06, 0.16 |
|  | Physical Comorbidiy | 0.03 | 0.06 | 0.58 | 0.57 | -0.08, 0.15 |
|  | Psychiatric Comorbidity | -0.05 | 0.06 | -0.93 | 0.35 | -0.16, 0.06 |
|  | Suicide Attempt | 0.07 | 0.06 | 1.16 | 0.25 | -0.05, 0.18 |
|  | Age at First Appointment | -0.02 | 0.14 | -0.11 | 0.91 | -0.29, 0.26 |
|  | Age at Initial Medication | -0.01 | 0.08 | -0.16 | 0.88 | -0.19, 0.15 |
|  | Number of Hospitalizations | -0.05 | 0.10 | -0.50 | 0.62 | -0.26, 0.15 |
| **Agreeableness** | (Intercept) | 0.12 | 0.09 | 1.26 | 0.21 | -0.06, 0.30 |
|  | Age | 0.33 | 0.20 | 1.60 | 0.11 | -0.07, 0.73 |
|  | Marital Status | -0.10 | 0.13 | -0.74 | 0.46 | -0.36, 0.16 |
|  | Years of Education | 0.15 | 0.09 | 1.73 | 0.08 | -0.02, 0.32 |
|  | Diagnosis | -0.14 | 0.07 | -2.03 | 0.04 | -0.28, -0.01 |
|  | Age at Illness Onset | 0.09 | 0.16 | 0.56 | 0.58 | -0.23, 0.41 |
|  | HAMD Score | -0.11 | 0.06 | -1.80 | 0.07 | -0.24, 0.01 |
|  | Age at First Hospitalization | -0.03 | 0.09 | -0.37 | 0.71 | -0.21, 0.14 |
|  | Number of Depressive Episodes | 0.04 | 0.04 | 0.90 | 0.37 | -0.05, 0.12 |
|  | Cumulative Depressed Days | -0.11 | 0.05 | -2.05 | 0.04 | -0.22, -0.01 |
|  | Whether Medicated | -0.14 | 0.09 | -1.61 | 0.11 | -0.31, 0.03 |
|  | Trauma History | 0.03 | 0.08 | 0.34 | 0.73 | -0.13, 0.18 |
|  | Family History of Psychiatric Disorsder | -0.03 | 0.05 | -0.66 | 0.51 | -0.14, 0.07 |
|  | Physical Comorbidiy | 0.03 | 0.06 | 0.54 | 0.59 | -0.09, 0.16 |
|  | Psychiatric Comorbidity | 0.06 | 0.06 | 1.00 | 0.32 | -0.06, 0.18 |
|  | Suicide Attempt | 0.09 | 0.06 | 1.50 | 0.13 | -0.03, 0.21 |
|  | Age at First Appointment | -0.05 | 0.16 | -0.31 | 0.76 | -0.36, 0.26 |
|  | Age at Initial Medication | 0.09 | 0.08 | 1.02 | 0.31 | -0.08, 0.25 |
|  | Number of Hospitalizations | 0.20 | 0.10 | 2.08 | 0.04 | 0.01, 0.39 |
| **Neuroticism** | (Intercept) | 0.05 | 0.09 | 0.55 | 0.58 | -0.13, 0.23 |
|  | Age | -0.28 | 0.22 | -1.32 | 0.19 | -0.71, 0.14 |
|  | Marital Status | -0.15 | 0.14 | -1.02 | 0.31 | -0.40, 0.14 |
|  | Years of Education | -0.09 | 0.10 | -0.89 | 0.37 | -0.29, 0.11 |
|  | Diagnosis | 0.06 | 0.07 | 0.77 | 0.44 | -0.09, 0.20 |
|  | Age at Illness Onset | -0.01 | 0.19 | -0.03 | 0.97 | -0.37, 0.36 |
|  | HAMD Score | 0.26 | 0.06 | 4.46 | **<0.001** | 0.15, 0.38 |
|  | Age at First Hospitalization | -0.07 | 0.13 | -0.50 | 0.62 | -0.32, 0.19 |
|  | Number of Depressive Episodes | 0.08 | 0.05 | 1.80 | 0.07 | -0.01, 0.18 |
|  | Cumulative Depressed Days | 0.03 | 0.07 | 0.46 | 0.65 | -0.11, 0.18 |
|  | Whether Medicated | -0.03 | 0.08 | -0.42 | 0.68 | -0.19, 0.12 |
|  | Trauma History | -0.02 | 0.06 | -0.27 | 0.79 | -0.13, 0.10 |
|  | Family History of Psychiatric Disorsder | -0.01 | 0.05 | -0.28 | 0.78 | -0.11, 0.09 |
|  | Physical Comorbidiy | 0.03 | 0.06 | 0.44 | 0.66 | -0.09, 0.14 |
|  | Psychiatric Comorbidity | -0.03 | 0.06 | -0.44 | 0.66 | -0.14, 0.09 |
|  | Suicide Attempt | -0.09 | 0.06 | -1.50 | 0.13 | -0.21, 0.03 |
|  | Age at First Appointment | 0.23 | 0.16 | 1.45 | 0.15 | -0.08, 0.55 |
|  | Age at Initial Medication | 0.05 | 0.07 | 0.76 | 0.45 | -0.08, 0.18 |
|  | Number of Hospitalizations | -0.04 | 0.13 | -0.33 | 0.74 | -0.30, 0.21 |

Note: CI = confidence interval. Bolded p-values indicate statistical significance at p < 0.05.

**Supplementary Table 7: Standardized Path Coefficients from the Structural Equation Model Predicting Diagnosis and Trait Associations**

| **Pathway** | **Std. β (Std.all)** | **Standard Error** | **p value** |
| --- | --- | --- | --- |
| Physical Comorbidities → Openness | 0.11 | 1.07 | 0.07 |
| Age → Conscientiousness | 0.28 | 0.03 | **0.00** |
| Years of Education → Conscientiousness | 0.23 | 0.09 | **0.00** |
| Psychiatric Comorbidities → Conscientiousness | -0.09 | 0.39 | 0.13 |
| Age → Agreeableness | 0.26 | 0.03 | **0.00** |
| Years of Education → Agreeableness | 0.19 | 0.08 | **0.00** |
| HAMD score → Neuroticism | 0.20 | 0.03 | **0.00** |
| Age →Years of Education | 0.14 | 0.01 | **0.00** |
| Conscientiousness → Diagnosis | 0.00 | 0.01 | 0.25 |
| Agreeableness → Diagnosis | 0.00 | 0.02 | 0.19 |
| Openness → Diagnosis | 0.12 | 0.01 | **0.00** |
| Extraversion → Diagnosis | 0.02 | 0.02 | 0.18 |
| Neuroticism → Diagnosis | 0.03 | 0.02 | 0.58 |
| Psychiatric Comorbidities → Diagnosis | 0.34 | 0.19 | **0.03** |
| Age → Diagnosis | -0.27 | 0.01 | **0.00** |
| Years of Education → Diagnosis | -0.12 | 0.03 | **0.08** |

**Supplementary Table 8: Associations Between Big Five Personality Traits and Clinical Features in Bipolar Disorder**

|  | **Variable** | **Coefficient** | **Standard Error** | **Lower CI** | **Upper CI** | **t value** | **p value** |
| --- | --- | --- | --- | --- | --- | --- | --- |
| **Openness to Experience** | Constant | 0.00 | 0.06 | -0.12 | 0.12 | 0.00 | 1.00 |
|  | Age | 0.20 | 0.22 | -0.23 | 0.63 | 0.92 | 0.36 |
|  | Marital Status | -0.04 | 0.09 | -0.23 | 0.14 | -0.48 | 0.63 |
|  | Years of Education | 0.24 | 0.07 | 0.11 | 0.37 | 3.68 | **0.00** |
|  | Bipolar Subtype | 0.09 | 0.07 | -0.05 | 0.23 | 1.30 | 0.20 |
|  | Age at Illness Onset | -0.08 | 0.13 | -0.34 | 0.18 | -0.62 | 0.53 |
|  | HAMD Score | -0.23 | 0.07 | -0.36 | -0.10 | -3.51 | **0.00** |
|  | Age at First Hospitalization | -0.13 | 0.12 | -0.37 | 0.11 | -1.04 | 0.30 |
|  | Number of Depressive Episodes | 0.09 | 0.06 | -0.04 | 0.22 | 1.41 | 0.16 |
|  | Cumulative Depressed Days | 0.02 | 0.07 | -0.11 | 0.15 | 0.27 | 0.78 |
|  | Whether Medicated | -0.08 | 0.09 | -0.26 | 0.10 | -0.84 | 0.40 |
|  | YMRS Scores | 0.06 | 0.07 | -0.08 | 0.19 | 0.81 | 0.42 |
|  | Age at First Appointment | -0.27 | 0.18 | -0.63 | 0.09 | -1.48 | 0.14 |
|  | Age at Initial Medication | -0.01 | 0.09 | -0.18 | 0.17 | -0.06 | 0.95 |
|  | Number of Hospitalizations | 0.06 | 0.12 | -0.17 | 0.30 | 0.52 | 0.60 |
|  | Suicide Attempt | -0.02 | 0.06 | -0.14 | 0.10 | -0.37 | 0.71 |
|  | Physical Comorbidity | 0.19 | 0.07 | 0.06 | 0.32 | 2.78 | **0.01** |
|  | Family History of Psychiatric Disorder | 0.09 | 0.06 | -0.03 | 0.21 | 1.41 | 0.16 |
|  | Trauma History | -0.05 | 0.06 | -0.18 | 0.08 | -0.77 | 0.44 |
| **Conscientiousness** | Constant | 0.00 | 0.06 | -0.12 | 0.12 | 0.00 | 1.00 |
|  | Age | 0.39 | 0.23 | -0.06 | 0.84 | 1.71 | 0.09 |
|  | Marital Status | 0.12 | 0.10 | -0.07 | 0.31 | 1.23 | 0.22 |
|  | Years of Education | 0.24 | 0.07 | 0.11 | 0.38 | 3.57 | 0.00 |
|  | Bipolar Subtype | 0.07 | 0.07 | -0.07 | 0.22 | 1.01 | 0.31 |
|  | Age at Illness Onset | 0.04 | 0.14 | -0.23 | 0.31 | 0.26 | 0.79 |
|  | HAMD Score | -0.14 | 0.07 | -0.27 | -0.01 | -2.06 | **0.04** |
|  | Age at First Hospitalization | -0.07 | 0.13 | -0.32 | 0.17 | -0.59 | 0.56 |
|  | Number of Depressive Episodes | 0.06 | 0.07 | -0.07 | 0.19 | 0.89 | 0.37 |
|  | Cumulative Depressed Days | 0.00 | 0.07 | -0.13 | 0.13 | 0.02 | 0.99 |
|  | Whether Medicated | -0.09 | 0.10 | -0.28 | 0.09 | -0.99 | 0.32 |
|  | YMRS Scores | 0.02 | 0.07 | -0.13 | 0.16 | 0.21 | 0.83 |
|  | Age at First Appointment | -0.31 | 0.19 | -0.69 | 0.06 | -1.63 | 0.11 |
|  | Age at Initial Medication | 0.11 | 0.09 | -0.07 | 0.30 | 1.22 | 0.23 |
|  | Number of Hospitalizations | 0.08 | 0.13 | -0.16 | 0.33 | 0.67 | 0.50 |
|  | Suicide Attempt | 0.03 | 0.06 | -0.10 | 0.15 | 0.39 | 0.69 |
|  | Physical Comorbidity | 0.06 | 0.07 | -0.08 | 0.20 | 0.84 | 0.40 |
|  | Family History of Psychiatric Disorder | 0.05 | 0.07 | -0.08 | 0.18 | 0.76 | 0.45 |
|  | Trauma History | 0.07 | 0.07 | -0.07 | 0.20 | 0.98 | 0.33 |
| **Extraversion** | const | 0.00 | 0.06 | -0.13 | 0.13 | 0.00 | 1.00 |
|  | Age | -0.05 | 0.23 | -0.51 | 0.40 | -0.23 | 0.82 |
|  | Marital Status | 0.16 | 0.10 | -0.03 | 0.35 | 1.61 | 0.11 |
|  | Years of Education | -0.03 | 0.07 | -0.16 | 0.11 | -0.40 | 0.69 |
|  | Bipolar Subtype | 0.02 | 0.07 | -0.12 | 0.17 | 0.31 | 0.75 |
|  | Age at Illness Onset | 0.08 | 0.14 | -0.19 | 0.36 | 0.60 | 0.55 |
|  | HAMD Score | -0.27 | 0.07 | -0.41 | -0.13 | -3.89 | **0.00** |
|  | Age at First Hospitalization | 0.03 | 0.13 | -0.23 | 0.28 | 0.21 | 0.83 |
|  | Number of Depressive Episodes | 0.04 | 0.07 | -0.09 | 0.18 | 0.61 | 0.54 |
|  | Cumulative Depressed Days | -0.10 | 0.07 | -0.24 | 0.04 | -1.45 | 0.15 |
|  | Whether Medicated | 0.04 | 0.10 | -0.15 | 0.23 | 0.44 | 0.66 |
|  | YMRS Scores | 0.13 | 0.07 | -0.02 | 0.27 | 1.74 | 0.08 |
|  | Age at First Appointment | 0.01 | 0.20 | -0.37 | 0.39 | 0.05 | 0.96 |
|  | Age at Initial Medication | 0.00 | 0.10 | -0.19 | 0.19 | -0.01 | 1.00 |
|  | Number of Hospitalizations | -0.01 | 0.13 | -0.26 | 0.24 | -0.06 | 0.95 |
|  | Suicide Attempt | 0.04 | 0.07 | -0.09 | 0.17 | 0.65 | 0.52 |
|  | Physical Comorbidity | 0.07 | 0.07 | -0.07 | 0.21 | 0.95 | 0.34 |
|  | Family History of Psychiatric Disorder | 0.06 | 0.07 | -0.08 | 0.19 | 0.82 | 0.41 |
|  | Trauma History | 0.03 | 0.07 | -0.10 | 0.17 | 0.46 | 0.65 |
| **Agreeableness** | Constant | 0.00 | 0.06 | -0.12 | 0.12 | 0.00 | 1.00 |
|  | Age | 0.21 | 0.22 | -0.23 | 0.65 | 0.93 | 0.35 |
|  | Marital Status | 0.05 | 0.09 | -0.13 | 0.24 | 0.54 | 0.59 |
|  | Years of Education | 0.13 | 0.07 | 0.00 | 0.26 | 1.99 | **0.05** |
|  | Bipolar Subtype | -0.13 | 0.07 | -0.27 | 0.01 | -1.83 | 0.07 |
|  | Age at Illness Onset | 0.11 | 0.14 | -0.15 | 0.38 | 0.84 | 0.40 |
|  | HAMD Score | -0.13 | 0.07 | -0.26 | 0.00 | -1.90 | 0.06 |
|  | Age at First Hospitalization | -0.15 | 0.12 | -0.39 | 0.10 | -1.19 | 0.24 |
|  | Number of Depressive Episodes | 0.07 | 0.07 | -0.06 | 0.19 | 1.02 | 0.31 |
|  | Cumulative Depressed Days | -0.03 | 0.07 | -0.16 | 0.10 | -0.49 | 0.62 |
|  | Whether Medicated | -0.16 | 0.09 | -0.34 | 0.03 | -1.67 | 0.10 |
|  | YMRS Scores | -0.10 | 0.07 | -0.24 | 0.04 | -1.42 | 0.16 |
|  | Age at First Appointment | -0.15 | 0.19 | -0.52 | 0.22 | -0.81 | 0.42 |
|  | Age at Initial Medication | 0.13 | 0.09 | -0.05 | 0.31 | 1.40 | 0.16 |
|  | Number of Hospitalizations | 0.29 | 0.12 | 0.05 | 0.53 | 2.35 | **0.02** |
|  | Suicide Attempt | 0.07 | 0.06 | -0.05 | 0.20 | 1.18 | 0.24 |
|  | Physical Comorbidity | 0.07 | 0.07 | -0.07 | 0.21 | 1.01 | 0.31 |
|  | Family History of Psychiatric Disorder | -0.02 | 0.06 | -0.14 | 0.11 | -0.24 | 0.81 |
|  | Trauma History | 0.00 | 0.07 | -0.13 | 0.13 | 0.05 | 0.96 |
| **Neuroticism** | Constant | 0.00 | 0.06 | -0.12 | 0.12 | 0.00 | 1.00 |
|  | Age | -0.25 | 0.22 | -0.68 | 0.18 | -1.15 | 0.25 |
|  | Marital Status | -0.15 | 0.09 | -0.33 | 0.03 | -1.65 | 0.10 |
|  | Years of Education | -0.13 | 0.07 | -0.25 | 0.00 | -1.92 | 0.06 |
|  | Bipolar Subtype | 0.03 | 0.07 | -0.11 | 0.16 | 0.40 | 0.69 |
|  | Age at Illness Onset | 0.02 | 0.13 | -0.24 | 0.28 | 0.17 | 0.87 |
|  | HAMD Score | 0.38 | 0.07 | 0.25 | 0.51 | 5.88 | **0.00** |
|  | Age at First Hospitalization | -0.16 | 0.12 | -0.40 | 0.08 | -1.31 | 0.19 |
|  | Number of Depressive Episodes | 0.07 | 0.06 | -0.06 | 0.19 | 1.04 | 0.30 |
|  | Cumulative Depressed Days | 0.04 | 0.07 | -0.09 | 0.17 | 0.57 | 0.57 |
|  | Whether Medicated | -0.02 | 0.09 | -0.20 | 0.16 | -0.22 | 0.83 |
|  | YMRS Scores | 0.02 | 0.07 | -0.11 | 0.16 | 0.31 | 0.76 |
|  | Age at First Appointment | 0.33 | 0.18 | -0.03 | 0.69 | 1.81 | 0.07 |
|  | Age at Initial Medication | 0.04 | 0.09 | -0.13 | 0.22 | 0.47 | 0.64 |
|  | Number of Hospitalizations | 0.03 | 0.12 | -0.21 | 0.26 | 0.22 | 0.83 |
|  | Suicide Attempt | -0.10 | 0.06 | -0.22 | 0.02 | -1.61 | 0.11 |
|  | Physical Comorbidity | -0.03 | 0.07 | -0.16 | 0.10 | -0.41 | 0.68 |
|  | Family History of Psychiatric Disorder | -0.04 | 0.06 | -0.17 | 0.08 | -0.68 | 0.50 |
|  | Trauma History | -0.01 | 0.06 | -0.14 | 0.12 | -0.14 | 0.89 |

Note: CI = confidence interval. Bolded p-values indicate statistical significance at p < 0.05.

**Supplementary Table 9: Logistic Regression Analysis of Clinical Predictors of Bipolar Disorder Diagnosis**

| **Variable** | **Estimate** | **Standard Error** | **Odds Ratio** | **Lower CI** | **Upper CI** | **t value** | **p value** |
| --- | --- | --- | --- | --- | --- | --- | --- |
| **(Intercept)** | 4.31 | 1.13 | 74.67 | 8.83 | 760.95 | 3.81 | <0.001 |
| **Age** | -0.02 | 0.04 | 0.98 | 0.91 | 1.06 | -0.52 | 0.60 |
| **Years of Education** | -0.10 | 0.05 | 0.90 | 0.81 | 1.00 | -1.96 | **0.05** |
| **Marital Status** | 0.13 | 0.51 | 1.14 | 0.43 | 3.23 | 0.26 | 0.79 |
| **HAMD Score** | -0.02 | 0.02 | 0.98 | 0.94 | 1.03 | -0.79 | 0.43 |
| **Cumulative Depressed Days** | 0.00 | 0.00 | 1.00 | 1.00 | 1.00 | -4.50 | <0.001 |
| **Whether Medicated** | 0.83 | 0.74 | 2.30 | 0.51 | 9.71 | 1.12 | 0.26 |
| **Age at Illness Onset** | -0.16 | 0.05 | 0.85 | 0.77 | 0.93 | -3.53 | **<0.001** |
| **Age at First Hospitalization** | -0.08 | 0.04 | 0.92 | 0.85 | 1.00 | -2.05 | **0.04** |
| **Number of Depressive Episodes** | -0.01 | 0.01 | 0.99 | 0.97 | 1.02 | -0.69 | 0.49 |
| **Trauma History** | -1.43 | 1.21 | 0.24 | 0.02 | 2.31 | -1.18 | 0.24 |
| **Family History of Psychiatric Disorder** | 0.35 | 0.53 | 1.42 | 0.53 | 4.29 | 0.66 | 0.51 |
| **Physical Comorbidity** | 0.01 | 0.41 | 1.01 | 0.46 | 2.33 | 0.02 | 0.98 |
| **Psychiatric Comorbidity** | 0.77 | 0.24 | 2.15 | 1.38 | 3.61 | 3.16 | **<0.001** |
| **Suicide Attempt** | 1.25 | 0.71 | 3.48 | 0.98 | 16.93 | 1.75 | 0.08 |
| **Age at First Appointment** | 0.10 | 0.04 | 1.10 | 1.02 | 1.19 | 2.55 | **0.01** |
| **Age at Initial Medication** | -0.03 | 0.02 | 0.97 | 0.93 | 1.01 | -1.65 | 0.10 |
| **Number of Hospitalizations** | 2.02 | 1.02 | 7.56 | 1.29 | 73.33 | 1.99 | **0.05** |

Note: CI = confidence interval. Bolded p-values indicate statistical significance at p < 0.05.
